# Dysregulation of Human Somatic piRNA Expression in Parkinson’s Disease Subtypes and Stages

**DOI:** 10.3390/ijms23052469

**Published:** 2022-02-23

**Authors:** Tianjiao Zhang, Garry Wong

**Affiliations:** Faculty of Health Sciences, University of Macau, Taipa, Macau SAR 999078, China; cb42277@connect.um.edu.mo

**Keywords:** piRNA, somatic piRNA, Parkinson’s disease, biomarker, sPLS-DA

## Abstract

Piwi interacting RNAs (piRNAs) are small non-coding single-stranded RNA species 20–31 nucleotides in size generated from distinct loci. In germline tissues, piRNAs are amplified via a “ping-pong cycle” to produce secondary piRNAs, which act in transposon silencing. In contrast, the role of somatic-derived piRNAs remains obscure. Here, we investigated the identity and distribution of piRNAs in human somatic tissues to determine their function and potential role in Parkinson’s disease (PD). Human datasets were curated from the Gene Expression Omnibus (GEO) database and a workflow was developed to identify piRNAs, which revealed 902 somatic piRNAs of which 527 were expressed in the brain. These were mainly derived from chromosomes 1, 11, and 19 compared to the germline tissues, which were from 15 and 19. Approximately 20% of somatic piRNAs mapped to transposon 3′ untranslated regions (UTRs), but a large proportion were sensed to the transcript in contrast to germline piRNAs. Gene set enrichment analysis suggested that somatic piRNAs function in neurodegenerative disease. piRNAs undergo dysregulation in different PD subtypes (PD and Parkinson’s disease dementia (PDD)) and stages (premotor and motor). piR-has-92056, piR-hsa-150797, piR-hsa-347751, piR-hsa-1909905, piR-hsa-2476630, and piR-hsa-2834636 from blood small extracellular vesicles were identified as novel biomarkers for PD diagnosis using a sparse partial least square discriminant analysis (sPLS-DA) (accuracy: 92%, AUC = 0.89). This study highlights a role for piRNAs in PD and provides tools for novel biomarker development.

## 1. Introduction

piRNAs (piwi-interacting RNAs) form the largest class of small non-coding RNAs in animals and are characterized by their length distribution of 21–30 nucleotides, interaction with PIWI proteins, and 2′-O-methylation at the 3′end of the single-stranded mature product [1,2,3,4]. To date, the most vital function of piRNAs known is the formation of a piRNA-PIWI silencing complex, which suppresses the expression of transposons in germline cells [5,6]. Because of the ubiquitous epigenetic reprogramming in the germline, transposons may transform their silent status to activation and subsequently alter their chromosome position or propagate, leading to a significant increased risk of genomic instability [7,8,9]. The deficiency of the piRNA-PIWI complex gives rise to the activation of transposons and causes sterility [10,11,12]. In animal models, the piRNA-PIWI complex suppresses transposons by depositing repressing genetic biomarkers (usually H3K9me3) at a transcriptional level or by utilizing PIWI-dependent cleavage of target mRNAs at a post-transcriptional level [13,14,15]. However, in mammalian germ cells, most pre-pachytene piRNAs appear to be within coding regions or are 3′ UTR derived [16,17]. The most abundant piRNA population, pachytene piRNAs, also show lack of transposon sequences [17,18,19]. The widespread existence and high expression level of non-transposon recognizing piRNAs suggest additional functions beyond transposon-silencing [20]. Previous studies have revealed that Aub (homolog of Piwi in *Drosophila*), a piRNA binding complex, increases the translation of self-renewal and differentiation factors in germ stem cells, as well as stabilize specific mRNAs by a piRNA-guided pathway [13,21]. Recently, another study suggested that a MIWI (homolog of Piwi in mouse)/piRNA/elF3f/HuR super-complex contains a translation-activating function in spermiogenesis [15].

In the germline, piRNAs’ biogenesis requires the ping-pong cycle for amplification of secondary piRNAs and is characterized by a 5′ U bias of primary piRNAs [22,23]. The first 10 base-pairs of primary piRNAs and their corresponding secondary piRNAs are complementary [22,23]. In contrast, the piRNA biogenesis pathway in somatic cells shows a distinct process [24,25,26]; however, the details remain elusive and controversial, especially in mammals. Many studies suggests somatic piRNAs exist and function in body regeneration [26,27], cancer [28], embryonic development [29], and neural development [30]. In *Homo sapiens*, transposons have the potential to increase the diversity of neurons [31]. For example, L1 can induce mosaicism in the neural genome [32]; and Alu is hypothesized to be related to human intelligence, cognition, and neurodegeneration [33]. Therefore, the transposon-repressing function of piRNAs seems to be necessary for neuronal systems. In addition, some studies also indicate that piRNAs function as gene expression regulators in somatic cells [34,35,36]. Many studies have found piRNAs to participate in bioprocesses in lower eukaryotes, including axon regeneration [37], foraging behavior [38], and memory [30,39,40,41]. The somatic piRNAs in the mammalian central nervous system were identified in mouse hippocampus and related to spine morphogenesis [42].

Parkinson’s disease (PD) is the second most common neurodegenerative disease with the most consistent neuropathological feature, the gradual reduction of dopaminergic neurons in substantia nigra pars compacta [43,44,45]. At least nine risk factors independently modify risk for PD, of which three decrease (coffee consumption, smoking, and physical activity) and six increase (family history of PD, dyspepsia, exposure to pesticides, oils, metals, and anesthesia) risk [44,46]. PD patients present with motor symptoms that typically include akinesia, bradykinesia, tremor, and rigidity but also may have gait disturbance, impaired handwriting, and speech deficits [47]. Non-motor symptoms include sensory deficits (hyposmia and impaired color vision), neuropsychiatric and neurological features (hallucinations, pain, anxiety, depression, early cognitive dysfunction, and dementia), sleep disturbance and bladder hyper-reflexia, and pain [48]. Some symptoms may overlap, for example, while visual and auditory hallucinations are known, olfactory hallucinations have been found in up to 11.3% of patients [49]. Apart from the substantia nigra, selective deterioration of neurons also to some extent exists in other brain regions, including the amygdala, basal nucleus of Meynert, hypothalamus, hippocampus, temporal cortex, cingulate cortex, and prefrontal cortex, which usually varies with different subtypes, stages, and symptoms [50,51]. Another remarkable characteristic of PD is the presence of Lewy bodies, which is the consequence of aberrant protein aggregation, mitochondria damage, lysosome dysfunction, and inflammation [52,53,54]. Studies have established that Lewy bodies consists of aggregated α-synuclein, [55], are facilitated by mitophagy and PINK1 [56,57], develop when the lysosome is unable to break down aggregating protein [58], and involve inflammation particularly through microglia [59,60]. A previous study indicated piRNAs were dysregulated in neuronal cells derived from induced pluripotent stem cells of sporadic PD patients compared to controls [61]. In the study, SINE and LINE-derived piRNAs were downregulated. In addition, two studies found different expression patterns of piRNAs in amyotrophic lateral sclerosis (ALS) and tauopathies [62,63]. In the ALS study, a *Drosophila* Cabeza (homolog of FUS causing ALS) knockdown model demonstrated increased pre-piRNA but lower mature piRNA levels [62]. In the tauopathy model, *Drosophila* was used again via neuron specific overexpression of tauR406W, a mutant form of tau. The animals showed locomotor deficits as well as neuronal degeneration where the RNAs from the head displayed 50 increased and 60 decreased transposable elements [63]. These investigators further validated their findings via observing increased transcripts of the retroviral class of transposable elements in human AD tissues [63]. These results suggest that piRNAs may be potential biomarkers for age-related neurodegenerative disease, including PD. Nonetheless, existing knowledge concerning piRNAs in PD remains obscure. Therefore, deeper and more accurate knowledge on the identity, abundance, distribution, and differences of piRNAs in neuronal tissues will help to inform and provide evidence of piRNAs as playing an essential role in neurodegenerative disease processes.

The piRNA-related discoveries in human somatic tissues are limited [64]. Moreover, some somatic piRNAs investigated by related research appear ambiguous as their alignment positions vastly overlap with other ncRNAs (e.g., tRNAs, rRNAs, and snoRNAs) [28,42,65,66], which suggests these somatic piRNAs might be overestimated as they are probably not bona fide piRNAs. In this study, we analyzed high-throughput small RNA sequencing data from several datasets downloaded from the GEO database with accession numbers and references provided therein. We used a relatively strict piRNA identification method by filtering other annotated small ncRNAs first and then annotated the rest of the sequences by a piRNA annotation database. Then, we analyzed the expression patterns and characteristics of germline piRNAs in tissue-specific somatic cells. To investigate piRNAs in PD tissues, we performed a comparative study of PD patients and controls and evaluated piRNAs’ functional role in disease onset and progression. Our investigation identifies a small but appreciable number of somatic piRNAs that exist in somatic cells and are dysregulated in human PD tissues. Furthermore, we identify the nature and potential gene targets of these piRNAs and propose a subset as disease biomarkers.

## 2. Materials & Methods

### 2.1. Workflow

In order to identify and compare piRNA expression patterns in germline tissues and somatic tissues, we built an analysis workflow. The workflow of the study includes pre-processing steps (quality control (QC), adapter trimming, and length filtering), annotation of piRNAs (mapping and annotation based on piRNA database). Next, tissue-specific piRNA features (chromosome distribution, genomic context, length distribution, and ping-pong cycle) were investigated. For the comparison study between PD and the control, we implemented differential expression analysis, followed by gene enrichment analysis for piRNAs-aligned to genes/predicted targets. Finally, classifiers were used to differentiate between the control and PD.

### 2.2. Collection of High-Throughput Small RNA Sequencing Datasets from GEO

We collected germline and somatic tissue small RNA sequencing datasets from GEO [67]. A previous study demonstrated that small RNA sequencing captures and sequences small RNAs with moderate-to-low-abundance while able to detect modest expression differences across samples [68]. We obtained the piRNA expression patterns in various tissues by analyzing their corresponding small RNA sequencing datasets. The description of datasets used for the study is provided in Table 1.

### 2.3. Pre-Processing and piRNAs Annotation

**Figure 1:**
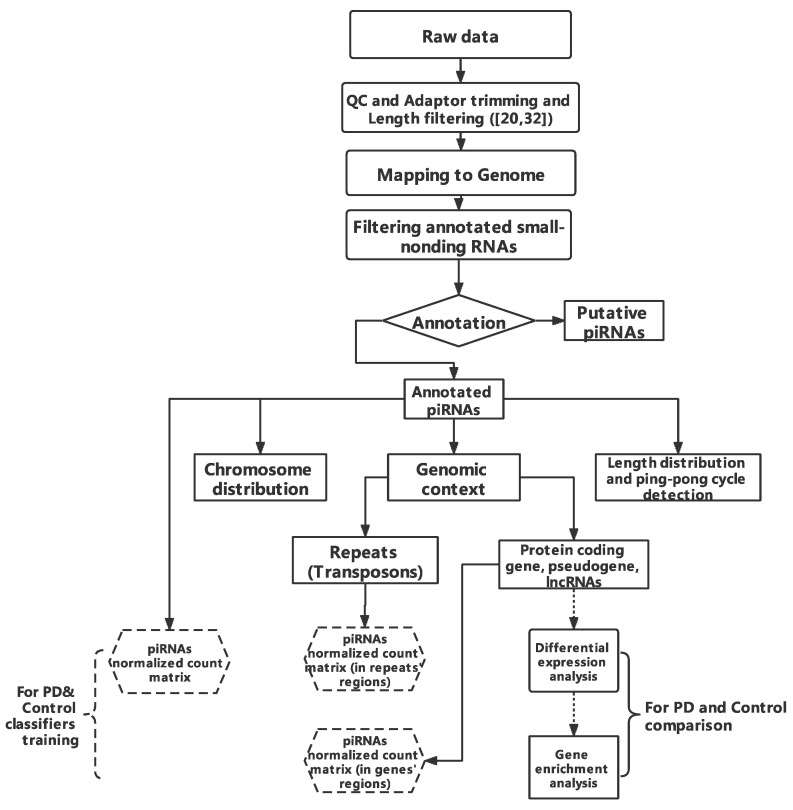
Workflow of this study. The raw data length filtered (20,32) is shown in brackets.

Sequenced raw data were adaptor trimmed and size filtered (20–32 nucleotides) by cutadapt (v2.8) [82]. We then aligned the processed sequencing data to hg38 Human Genome assembly [83] by bowtie (v1.2.3) [84] with one mismatch tolerance, and alignments with more than 50 distinct positions restrained. To filter other small non-coding RNAs (rRNAs, tRNAs, miRNAs, scaRNAs, snoRNAs, miscRNAs, scRNAs, sRNAs, and snRNA) in our datasets, we created an aggregated small non-coding RNA annotation file based upon UCSC [85], RefSeq [86], Gencode [87], DASHR [88], and Ensemble databases and annotations [83]. We excluded reads aligned to these other small non-coding RNAs from further investigation. We used featureCounts (v2.0.0) [89] and piRNA annotation file download from piRbase [90] to annotate piRNAs in the remainder of the reads. The reads annotated by piRBase were considered as annotated piRNAs in our study. The general workflow of the study is shown below as Figure 1.

### 2.4. Transposons and Transcripts Annotation

Repeats’ annotation was downloaded from UCSC repeat browser [91], while transcripts annotation (protein-coding, lncRNAs, pseudogenes) was obtained from Ensemble [83]. In our study, we classified LINE, SINE, LTR, and DNA in repeats as transposons and the rest of the repetitive sequence types, such as simple repeats, as other repeats.

### 2.5. Differential Analysis and Enrichment Analysis

We used the sample size, variance homogeneity of variance, and existence of outliers to construct differential analysis by the Wilcoxon test (piRNAs) or Deseq2 (v1.30.1) [92]. Combat-seq algorithm [93] was used to correct batch effects when needed. We implemented gene ontology (GO) and Kyoto Encyclopedia of Genes and Genomes (KEGG) pathway enrichment analysis by a web-based resource g: Profiler [94].

### 2.6. Classifier Construction

Sparse partial least squares discriminant analysis (sPLS-DA) [95,96,97] was used to construct control and PD classifiers. For each classifier, we used expression counts data (piRNAs, piRNAs in each repeat region, and piRNAs in each gene region) normalized by library size to train the classifier. In each training process, we first evaluated the performance of sPLS-DA by component numbers from one to ten, using leave-one-out cross-validation [98,99] to obtain optimized component numbers. We then used the grid-search method to access the optimal number of variables to select on each component for a further sparse partial least squares discriminant analysis (sPLS-DA). Finally, we used accuracy and area under the curve (AUC) of the receiver operating characteristic curve (ROC) [100] to identify the quality of each classifier. We used package mixOmics [101] to complete the processes mentioned above.

## 3. Results

### 3.1. piRNA Expression in Somatic Cells and Comparison to Germline Cells

**Figure 2:**
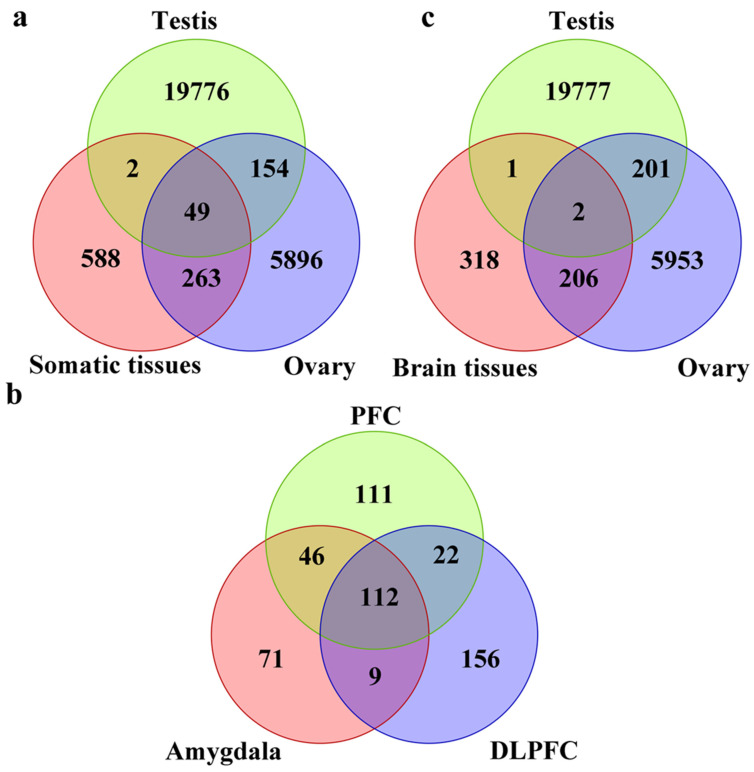
Venn plots of piRNAs identified from germline and somatic tissues. The number of piRNA types among somatic (dorsolateral prefrontal cortex, prefrontal cortex, amygdala, pancreas, liver, and knee synovial), germline tissues (testis, sperm, and ovary), and brain tissues (dorsolateral prefrontal cortex (DLPFC), prefrontal cortex (PFC), and amygdala) were compared. (**a**) Comparison between somatic and germline tissues. (**b**) Comparison between brain and germline tissues. (**c**) Comparison between different brain regions.

In order to characterize the properties of somatic piRNAs, we built a pipeline for extracting and identifying piRNAs from next generation sequencing (NGS) datasets. The pipeline is shown in (Figure 1). As the ovarian tissue dataset has a different experimental design and sequencing depth with others, piRNAs were considered expressed if reads were >2 in all samples or reads > 5 in 50% of samples, while for other tissues, the standard was reads > 2 in all samples or reads > 10 in 50%. We first compared piRNAs from somatic tissue to the testis and ovaries. The total number of piRNAs identified from the testis, ovary, and somatic tissue were 19981, 6363, and 902, respectively (Figure 2a). Consistent with previous studies, we identified a large number of testis piRNAs and a small number of somatic RNAs [102]. There were 49 common piRNAs between tissues studied, 51 between somatic and testis tissues, and 312 between somatic and ovaries, representing >30% of all somatic piRNAs. When the brain piRNAs were considered, the total number of piRNAs were 527 (Figure 2b). There was a dramatic reduction in piRNAs overlapping with testis, 3 in total, while 208 overlapped with ovaries. When we further compared brain regions, we observed that piRNAs were widely dispersed between the amygdala, prefrontal cortex, and dorsal lateral prefrontal cortex (Figure 2c). The expression pattern of somatic piRNA types appears to be tissue-specific with a large proportion of different piRNAs in the germline (testis, sperm, and ovary) (Figure 2a–c) (Supplementary Figure S1), which suggests that somatic piRNAs might function differently than those from germline piRNAs.

**Figure 3:**
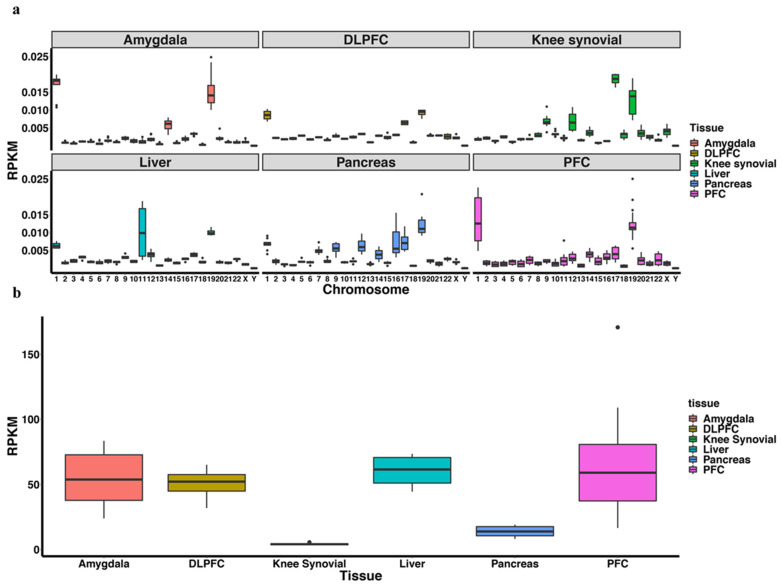
Chromosome distribution of piRNAs. (**a**) The distribution of piRNA reads mapped to the genome across different tissues was plotted. The *y*-axis represents RPKM which is calculated by $\frac{The\;number\;of\;reads\;mapped\;to\;chromosome\times 10^{6}}{The\;number\;of\;piRNAs\times length\;of\;the\;chromosome}$. A fraction count to each multi-alignment reads was assigned (each aligned-read carries 1/x count where x is the number of alignment positions of that read). (**a**) shows piRNA distribution in autosomes while (**b**) shows piRNA distribution in the mitochondria chromosome.

piRNAs are known to be expressed from specific loci in the genome. In order to identify the specific regions of the genome from where the piRNAs originated, we mapped the reads. Testis/sperm piRNAs display a pronounced chromosome 15 and 19 alignment preference, which is lost in the ovary (Figure 3a). Chromosome 19 alignment can also be observed in somatic tissue; however, there appears to be piRNA loci, such as chromosome 4 for ovary, 1 for brain tissues, liver, and pancreas, and 11 for liver (Figure 3a). Somatic piRNAs also show a high mitochondrial chromosome alignment rate among all three brain tissues studied and the liver in contrast to germline tissues (Figure 3b).

**Figure 4:**
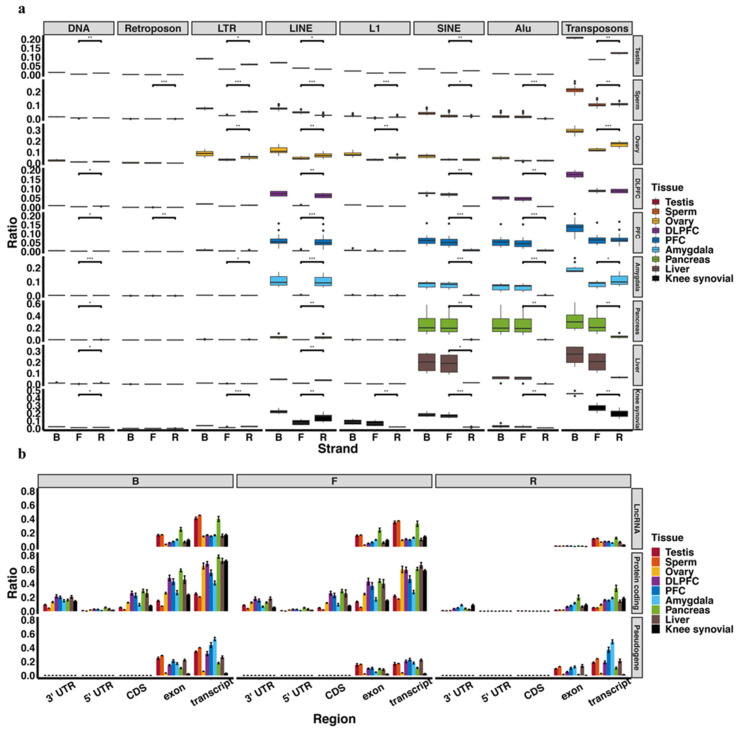
Genomic context of piRNAs. The percentage of annotated piRNAs reads mapped to transposons/transposon subtypes (**a**) and lncRNA/protein-coding genes/pseudogenes (**b**) of total annotated piRNA reads number. Ratio = reads number mapped to transposons (or lncRNA, protein-coding genes, pseudogenes)/total annotated piRNAs reads number. Symbols: *, *p* < 0.05; ** *p* < 0.01; *** *p* <0.001.

To determine whether somatic piRNAs were functioning via the well-established transposon suppressing pathway, we identified the transposon sequences aligned to the piRNAs. We unexpectedly detected a lower percentage (~20%) of piRNAs aligned to transposon sequences in both somatic tissues and germline tissues (Figure 4a). This lower percentage in germline was also found by other studies [103,104]. Unlike other tissues, amygdala and germline piRNAs contain more reverse-stranded repeat transposons than stranded. Considering the prevalent model related to piRNA-guided transposon silencing, where the PIWI-piRNA complex interacts with a nascent transposon transcript, a higher reverse-stranded piRNA percentage might indicate that repressing transposon expression is a vital function in both amygdala and germline tissues. In LINE-alignment piRNAs, somatic and ovarian piRNAs show a reverse-stranded preference, while testis and sperm piRNAs present the opposite. However, testis piRNAs show a reverse-stranded preference in SINE-alignment piRNAs, while somatic piRNAs are just the opposite (Figure 4a). Because LINE and SINE might have different impacts in somatic and germline tissues, their aligned-piRNAs might have varying regulatory functions.

We observed that testis and sperm piRNAs showed a higher percentage of lncRNA-alignment (Figure 4b). These are mainly mammalian pachytene piRNAs, which are primarily generated from transposon-depleted lncRNAs and thought to regulate genes required for male fertility [17]. We detected a higher ratio of protein-coding derived piRNAs, especially in somatic tissues, suggesting a function different that in germline cells. This phenomenon is partly contributed by some coding-gene (~25%) containing transposon sequences in the 3′UTR [105]. Some studies demonstrated that these genes have the potential to be controlled by PIWI and transposon-derived piRNA complex [106], but it is more likely that there are more protein-coding-derived piRNAs that exist across tissues. Currently the detailed function of coding-protein-derived piRNAs remains unknown.

We also identified many piRNAs aligned to pseudogenes, which is more evident in testis and sperm tissue as their lower protein-coding gene alignment rate (Figure 4b). A recent study suggested that some pseudogene-derived piRNAs have the potential to target and regulate their cognate protein-coding genes [106]. A more detailed study might thus unravel a regulation network among pseudogenes, piRNAs, and protein-coding genes [107].

**Figure 5:**
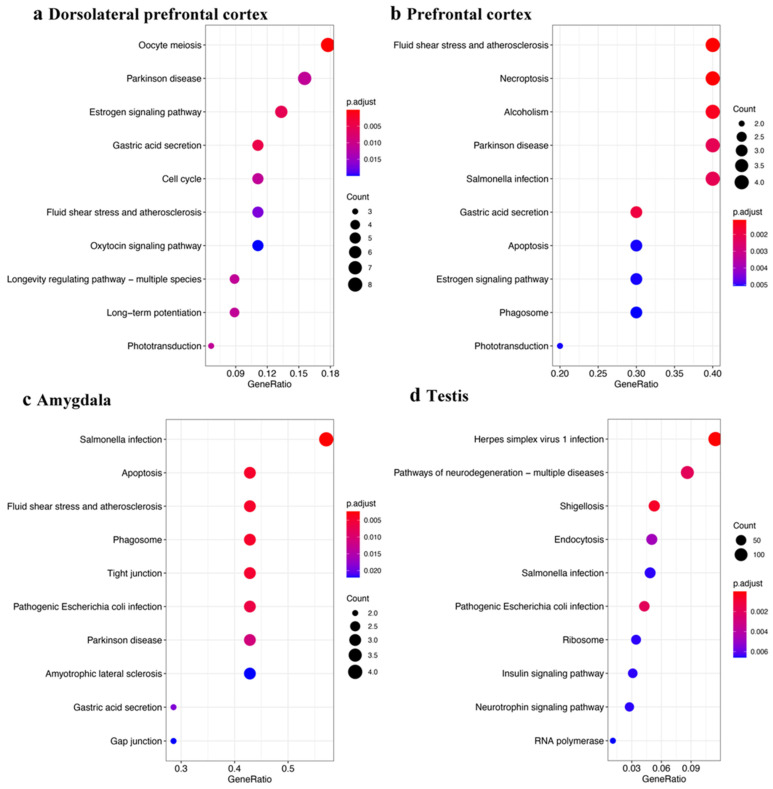
KEGG enrichment results for 3′UTR-piRNA alignment genes. KEGG enrichment analysis with protein-coding genes containing 3′UTR aligned by annotated piRNAs in brain tissues (**a**–**c**) and testis tissue (**d**). Gene ratio = Gene number in query list (piRNAs aligned in gene list)/Total gene number of specific pathway in database.

Next, to more clearly define a function for somatic piRNAs, we performed KEGG enrichment analysis with genes aligned by piRNAs at the 3′UTR region. Previous studies reported the existence of pre-pachytene piRNAs derived from 3′UTR of protein-coding genes in germline cells [17]. To our surprise, both brain tissue and testis tissue achieve enrichment results for “Parkinson’s disease” and “Pathways of Neurodegeneration–multiple diseases”, respectively (Figure 5). However, the function of these sense-piRNAs is indistinct. They might be transacting with their derived mRNAs by an unknown mechanism. When we used the transcript (including CDS, exon, 3′UTR, and 5′UTR) aligned by piRNAs to perform enrichment analysis of the three different brain tissues, testis, and ovary, all tissues identified an enrichment of neurodegeneration related genes.

**Figure 6:**
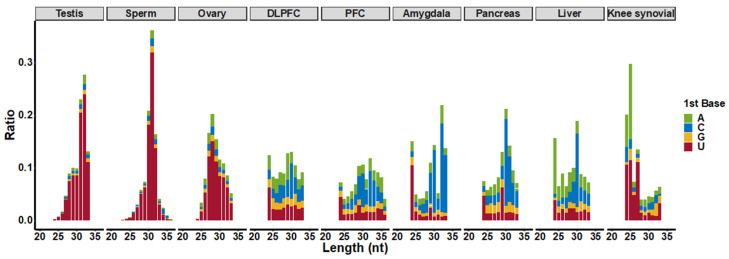
The length distribution of annotated piRNAs. The *y*-axis represents the ratio of piRNA reads with specific length/base to total annotated piRNAs reads in that tissue. Colors represent the first base of annotated piRNAs.

To further define attributes of piRNAs from somatic tissues, we characterized their length features. Somatic piRNAs show shorter and more evenly distributed lengths than germline piRNAs (Figure 6). According to the ping-pong cycle biogenesis pathway, tissues with significant ping-pong cycle signals synthesize piRNAs, which possess a 5′U bias and an A nucleotide bias at the 10th position. We found these attributes in testis, sperm, and ovary derived piRNAs from our analysis pipeline. However, we were not able to uncover evidence of ping-pong cycle biogenesis in somatic tissues based upon the distribution of mapped reads (Figure 6). We further confirmed this with an algorithm, pingpongpro [108], which also was not able to detect a ping-pong cycle signal in somatic tissues.

### 3.2. piRNAs in Parkinson’s Disease

**Figure 7:**
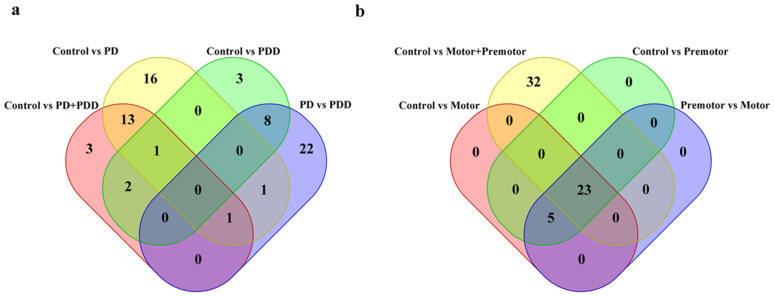
Venn plots of differently expressed piRNAs. Venn plots of differentially expressed piRNAs from prefrontal cortex (**a**) and amygdala (**b**). The Wilcoxon test was used to detect differently expressed piRNAs between different groups with *p*-value < 0.05. PD: Parkinson’s disease; PDD: Parkinson’s disease dementia.

To determine the significance of somatic piRNAs in human disease, we detected 296 piRNAs in the prefrontal cortex of which 20 piRNA expression levels were significantly different in PD; and 508 piRNAs in amygdala of which 55 piRNA expression levels were significantly different in PD (Figure 7a). Among these, one piRNA (piR-hsa-748391) was expressed differently in PD in both prefrontal cortex and amygdala tissues. Unfortunately, none of 3′UTR derived piRNAs from neurodegeneration-related genes mentioned above were among these differently expressed piRNAs. A target predicting method raised by a previous paper [109] showed 55 differently expressed piRNAs in amygdala target 20 proteins. Of these 20 proteins, MT-CO1 and MT-CO3 were included in the Parkinson’s disease KEGG enrichment result, while MT-CO1, MT-CO3, and GRIA4 was included in the Pathways of neurodegeneration-multiple diseases KEGG enrichment result. However, this stringent target prediction method predicted that none of the genes were targeted by piRNAs differently expressed in PD prefrontal cortex.

We also found five piRNAs were differently expressed among the control, premotor stage PD, and motor stage PD in amygdala but not in the control vs. PD (premotor plus motor stage) (Figure 7b). Among these, piR-hsa-131693 is predicted to target MT-CO3 and MT-CO3 related pseudogenes (MT-CO3, MTCO3P12, MTCO3P18, MTCO3P8, MTCO3P38, MTCO3P7, MTCO3P13, and MTCO3P22) and the expression of piR-has-131693 decreases as PD disease progresses in amygdala. In the prefrontal cortex, we also identified different piRNAs expression patterns in PD and PDD, although there were no significant enrichment results with their predicted target genes (Figure 7a). In PD SEVs, we found 17 differently expressed piRNAs, of which piR-hsa-2435261 was predicted to target TANGO2 and piR-hsa-1516701 was predicted to target JAK3. In PD LEVs, two differently expressed piRNAs were detected with no specific predicted target genes.

**Figure 8:**
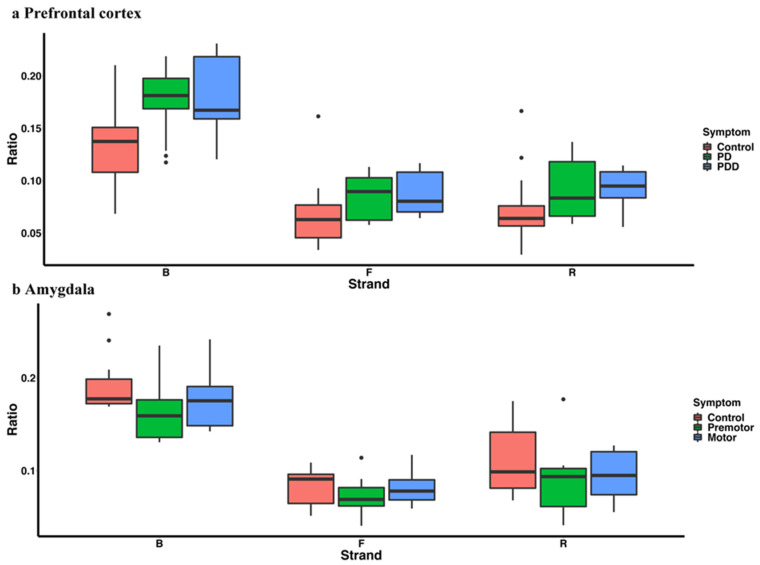
The percentage of annotated piRNAs reads mapped to transposons in prefrontal cortex (**a**) and amygdala (**b**) Ratio = reads number mapped to transposons (or subtypes)/total annotated piRNAs reads number.

**Figure 9:**
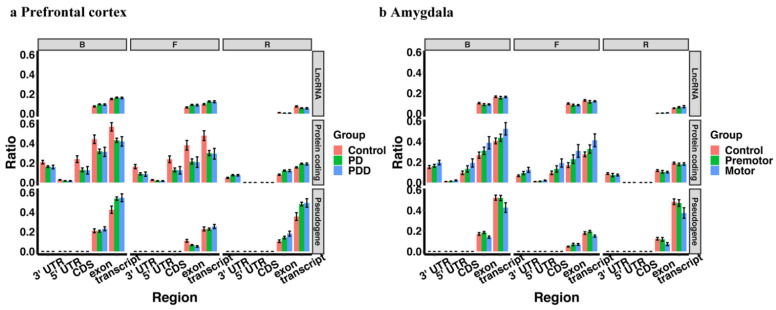
The percentage of annotated piRNAs reads mapped to transcripts in prefrontal cortex (**a**) and amygdala (**b**) Ratio = reads number mapped to lncRNA (or protein-coding genes, or pseudogenes)/total annotated piRNAs reads number.

We observed a higher percentage of transposon-derived piRNAs in the PD group in the prefrontal cortex (control vs. PD, control vs. PDD, control vs. PD plus PDD), but no significant difference between PD and PDD (Figure 8a). However, the transposon-derived piRNA expression difference between PD and control in amygdala is not significant (Figure 8b). In contrast, piRNAs in PD showed higher pseudogene alignment in the prefrontal cortex (Figure 9a), where the different piRNA-aligned pseudogenes were HSP90AA1 and EEF1A1-related pseudogenes. Interestingly, these two genes have been reported as PD-related genes and showed a downregulation trend in PD-affected tissues [110,111]. In amygdala, the overall piRNAs-aligned pseudogenes ratio is not different in Parkinson’s disease (Figure 9b), but some related pseudogene-aligned piRNAs, including HSP90, MTCO1, MTCO2, YWHAZ, GAPDH, and MTND were upregulated in the PD group.

**Figure 10:**
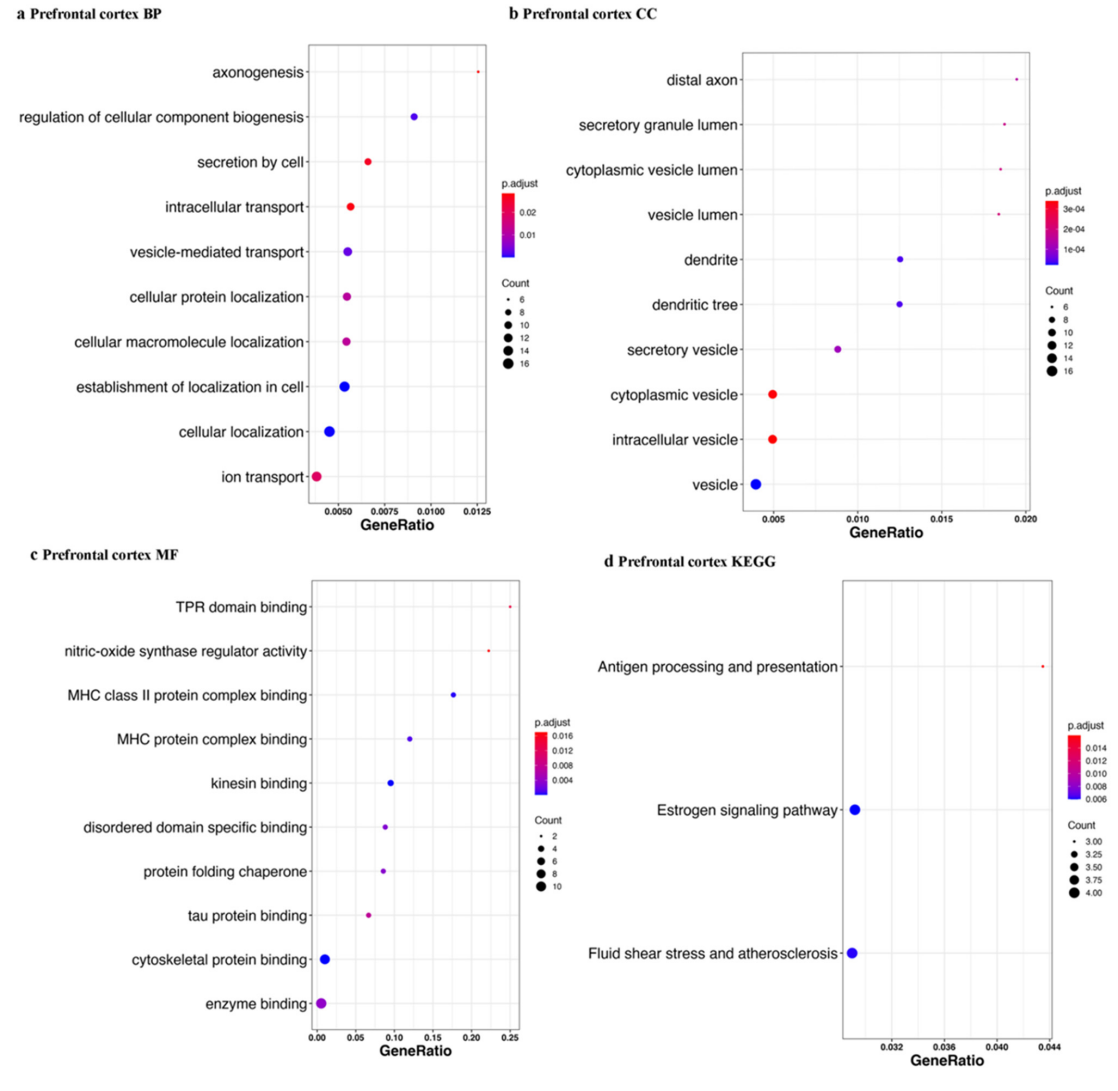

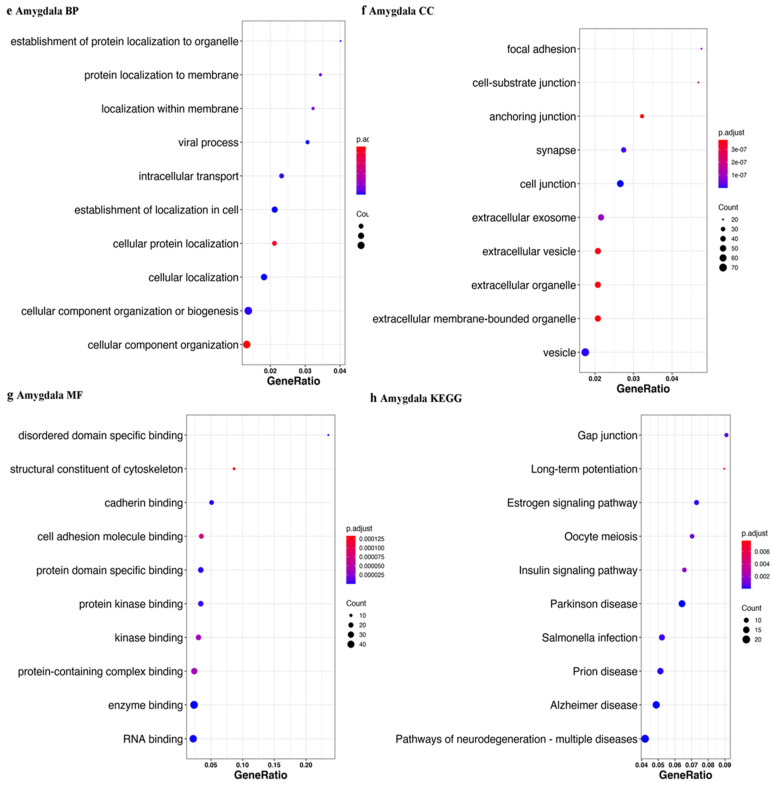
Gene set enrichment analysis for genes with different piRNAs-aligned rates. piRNAs-aligned genes was defined by a stringent standard (piRNAs-aligned number > 10 in > 50% of samples or > 2 in 100% of samples). The gene ontology and KEGG pathway enrichment analyses were established by genes with different piRNA aligned rates in PD and control groups. The top 10 results for each enrichment term: BP (biological process), CC (cellular component), MF (molecular function), and KEGG are shown. The tissues are indicated (**a**–**h**). Gene ratio = gene number in query list (piRNAs aligned in gene list)/total gene number of specific pathway in database.

We defined piRNAs-aligned genes with a stringent standard (piRNAs-aligned number > 10 in more than 50% samples or piRNAs-aligned number > 2 in all samples). Using differently expressed analysis (*p* < 0.05), we identified 24 genes with different piRNAs-alignment numbers between the control and PD in prefrontal cortex and 162 genes in amygdala. Figure 10 shows the top 10 results for each enrichment term. We also obtained Parkinson’s disease KEGG results in the amygdala tissue and prefrontal tissue with a relaxed standard (piRNAs-aligned number >2 in more than 10 samples) (data not shown). From the Parkinson’s disease KEGG enrichment result, five of differently expressed piRNAs-aligned genes (NDUFA4, CALM3, UCHL1, CALM2, and HSPA5) in the prefrontal cortex (15 genes) overlapped with differently expressed piRNAs-aligned genes in the amygdala (16 genes).

### 3.3. sPLS-DA of piRNAs Expression Level in Control and PD

**Figure 11:**
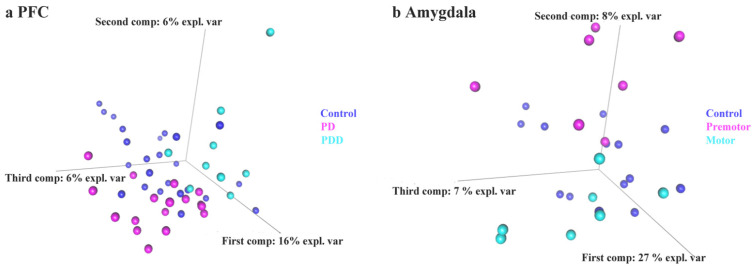
sPLS-DA of piRNA expression data. The 3D sPLS-DA plots for prefrontal cortex (**a**) and amygdala (**b**) piRNA expression levels are shown. The optimized components value of blood SEVs is 1, therefore was not presented as a 3d PLS-DA plot.

Lastly, we employed a sparse partial least square discriminant analysis, a supervised principal component analysis analog statistical method [95,97], to distinguish control and PD based on piRNAs expression levels. The leave-one-out cross-validation method was applied to construct 2-classes classifiers/3-classes classifiers (results are shown in Supplementary Table S1). These results show the potential that piRNAs can be biomarkers to separate PD from controls, suggesting piRNAs undergo perturbation in different PD subtypes (PD and PDD) and stages (premotor and motor). We also used piRNAs counts in piRNAs-aligned genes/repeats as indicators to train sPLS-DA classifiers, which showed validation performance improvement over the above piRNAs model to some extent. We also used a random forest algorithm and the results obtained were similar (data not shown). We also observed that a classifier based on piRNAs in blood extracellular vesicles achieve an excellent performace (accuracy: 92%, AUC = 0.89). The sPLS-DA based scatter plots for individuals show a clear separation (Figure 11).

## 4. Discussion

In this study, we identified piRNAs that display specific expression patterns across germline and somatic tissues. Compared with germline derived piRNAs, the overall abundance in somatic tissues was much lower but still appreciable. A previous study proposed that piRNA targeting has a greater correlation with binding energy compared to piRNA abundance, suggesting that piRNAs might act in a concentration-independent manner [112]. This supports the notion that somatic piRNAs can exert their functions with low expression levels. We found evidence for a ping-pong cycle biogenesis pathway signal in germline (testis, sperm, and ovary) but not in somatic tissues, which suggests a different piRNA generation pathway in the soma. The chromosomal loci from where piRNAs are generated showed some overlap between somatic and germline tissues; however, most somatic piRNAs were generated from loci distinct from germline cells.

While the existence of piRNAs from somatic tissues has been described, the function of these piRNAs remains unclear [107,113,114]. It could be hypothesized that somatic piRNAs function similarly to germline piRNAs, where the primary function is to repress transposons [115]. We found only 20% of piRNAs derived from transposon areas in both germline and somatic tissues. As somatic cells have more extensive epigenetic biomarkers than germline cells [116], the primary function of somatic piRNAs might be different from repressing transposons. Notably, transposon-derived somatic piRNAs have different expression levels and stranded preferences with transposon-derived germline piRNAs, suggesting that transposon repression may not be its only function.

Previous studies have shown evidence that piRNAs regulate protein expression and participate in many bioprocesses [117,118,119,120]. Some studies suggest that piRNAs repress gene expression by cleaving mRNAs of protein-coding genes [13,14,121,122]. Another study showed non-transposon-derived piRNAs have the potential to induce DNA methylation at non-transposon regions in a complementary manner in two human cell lines [123]. In addition, studies have indicated that piRNAs activate mRNA translation [15] and localize mRNA [124,125]. Conversely, some evidence has also shown that mRNAs act as a source of piRNAs, which in turn regulate their target mRNAs [22,126]. We hypothesized that piRNAs’ generation and function follow base-pairing rules, and we performed enrichment analysis with the genes aligned by piRNAs. The enrichment results showed neurodegeneration disease in both brain tissues (dorsolateral prefrontal cortex, prefrontal cortex, and amygdala) and germline tissues (testis and ovary), which suggested piRNAs may be involved in neurodegenerative processes. To further test this, we performed a comparative study of Parkinson’s disease in the prefrontal cortex and amygdala. The prefrontal cortex and amygdala were previously shown to be affected in Parkinson’s disease in a study using lesions with immunoreactions against α-synuclein and focusing on limbic and motor regions, and in PET imaging studies measuring blood flow changes, respectively [127,128]. Especially, amygdala was demonstrated to be damaged in the early stage and related to some incipient symptoms, such as anxiety determined by the association of anxiety symptoms and amygdala volume in 110 early stage patients [129]. Imaging studies have also addressed the link between anxiety and the amygdala in Parkinson’s disease especially highlighting the fear circuit [130]. We found that the piRNAs in Parkinson’s disease brains have a higher pseudogene alignment rate, while the cognate protein-coding genes of some of those pseudogenes showed neurodegeneration-related functions. Pseudogene-derived piRNAs have been proposed to suppress their cognate gene level in late spermatocytes [106]. Based on these results, we propose a pseudogenes-piRNAs protein-coding gene regulation network, where those pseudogenes-related piRNAs have the potential to regulate their cognate genes in brains.

Finally, we used piRNAs expression levels as features to construct sPLS-DA classifiers with the prefrontal cortex, amygdala, and blood extracellular vesicles [97]. The blood extracellular vesicle-related classifier is particularly interesting, as it has potential to be measured as a noninvasive biomarker in Parkinson’s disease [131,132,133,134,135] To our surprise, the classifier’s performance with small extracellular vesicle piRNAs was excellent (accuracy: 92%, AUC = 0.89). This compares favorably to previous studies where miRNAs were proposed as biomarkers and may further complement studies where the amount of exosomes, α-synuclein, or other proteins were proposed [134,136,137,138]. The variable importance in projection (VIP) score in sPLS-DA indicates a feature’s relevance and importance to the trained model. We found piR-has-92056, piR-hsa-150797, piR-hsa-347751, piR-hsa-1909905, piR-hsa-2476630, and piR-hsa-2834636 showed the highest relevance in our trained model. piR-hsa-92056 and piR-hsa-150797 are derived from SPPL3, which is a signal peptide peptidase and identified as a risk gene of PD in a GWAS study [139]. piR-hsa-2476630 is derived from DOK3, which inhibits lipopolysaccharide signaling in macrophages [140], while lipopolysaccharide causes chronic neuroinflammation and progressive neurodegeneration [141]. piR-has-347751 is derived from RHEB, which alleviates neurodegeneration and induces axon regrowth [142]. piR-hsa-2834636 is derived from HBA2, of which the mRNA is dysregulated in the frontal cortex of neurodegenerative disease [143]. While these associations suggest specific targets through which the piRNAs may act, validation of each specific biochemical step remains to be performed. Model organisms may thus help in this regard, and a study from our lab demonstrated dysregulation of specific miRNAs and piRNAs in a PD model [144]. Ultimately, the function piRNAs in blood extracellular vesicles might reveal information concerning cell–cell communication and disease progression.

While we were able to detect specific piRNAs in somatic tissue and demonstrate their dysregulation, the abundance is very small, especially compared to germline tissues. Moreover, we were not able to detect evidence of an amplification system or biological process to produce secondary piRNAs. How these modest levels of piRNAs affect their cognate genes remains to be determined. Recent studies suggest that multiple piRNAs may target a specific mRNA or have broad targeting capacity [109,112]. Thus, the predicted targets of the piRNAs in this study may require further verification and/or refinement. Furthermore, while the notion of dysregulated piRNAs from blood is attractive, their biological origins, such as from brain or other tissues, remain elusive. Previous work on the role of inflammatory processes in neurodegeneration suggests a blood-based pathway but requires additional investigation [145]. Finally, while recent studies have linked transposon expression in the brain to Alzheimer’s disease processes, the role of these sequences in PD is largely unknown [146,147].

## 5. Conclusions

In summary, we identified and characterized somatic piRNAs from a multitude of non-germline tissues. Their distribution and source of biogenesis appeared to differ from germline tissues. Their function, based upon cognate RNA targets appeared to be different as well. Surprisingly, their gene targets were enriched for neurodegenerative disease and specific genes identified highlighted pseudogenes related to Parkinson’s disease. The results presented here provide insights into the potential role of piRNAs in human neurodegenerative disorders.

## Figures and Tables

**Table 1:** Description of small RNA datasets curated for the analysis.

Tissue	Accession	Sample Size	Library Size	References
Testis	PRJNA196749	3	6.84–25.30 million	[69]
Sperm	PRJNA564759	102	0.36–1.63 million	[70]
Ovary	PRJNA272542	12 Note: 4 adult ovaries; 4 ovaries from 1st trimester embryos; 4 ovaries from 2st trimester embryos	13.3–15.8 million	[71]
Dorsolateral Prefrontal Cortex (DLPFC)	PRJNA185476	4	6–12.25 million	[72]
Pancreas	PRJNA490335	3	12.2–12.5 million	[73]
Knee synovial tissues	PRJNA389258	10	8.39–17.21 million	[74]
Liver	PRJNA246372	4	5.83–40.70 million	[75]
Prefrontal cortex (PFC)	PRJNA295431 PRJNA272617	26 (Parkinson’s disease) Note: including 17 Parkinson’s disease (PD); 9 Parkinson’s disease with dementia (PDD) 25 (Control)	6.09–33.40 million	[76,77,78]
Amygdala	PRJNA381204	14 (Parkinson’s disease) Note: including 7 premotor stage; 7 motor stage 14 (Control)	13.5–17.7 million	[79,80]
Blood (extracellular vesicles)	PRJNA655240	9 (Parkinson’s disease) Note: in each sample, both large (LEV) and small extracelluar vesciles (SEVs) were tested 6 (Control) Note: in each sample, both large (LEV) and small extracelluar vesciles (SEVs) were tested	2.37–30.7 million	[81]

## Data Availability

Data were obtained from the GEO database https://www.ncbi.nlm.nih.gov/geo/ accessed on 14 January 2022. All data used in this study can be obtained from accession numbers provided in Table 1.

## Supplementary Materials

The following supporting information can be downloaded at: https://www.mdpi.com/article/10.3390/ijms23052469/s1.

## Abbreviations

ALS	amyotrophic lateral sclerosis
AUC	area under curve
DLPFC	dorsolateral prefrontal cortex
GEO	Gene Expression Omnibus
GO	gene ontology
KEGG	Kyoto Encyclopedia of Genes and Genomes
LEVs	large extracellular vesicles
LINE	long interspersed nuclear element
lncRNA	long non-coding RNA
LTR	Long terminal repeat
miRNA	microRNA
miscRNA	miscellaneous RNA
ncRNA	non-coding RNA
NGS	next generation sequencing
PD	Parkinson’s disease
PDD	Parkinson’s disease dementia
PFC	prefrontal cortex
piRNA	piwi-interacting RNAs
PIWI	P-element induced wimpy testis in *Drosophila*
ROC	receiver operating characteristic
rRNA	ribosomal RNA
scaRNA	small cajal body-specific RNA
scRNA	small conditional RNA
SEVs	small extracellular vesicles
SINE	short interspersed nuclear element
snoRNA	small nucleolar RNA
snRNA	small nuclear RNA
sPLS-DA	sparse partial least square discriminant analysis
sRNA	bacterial small RNA
tRNA	transfer RNA
UTR	untranslated region

## References

[1] Weick E.M., Miska E.A. (2014). piRNAs: From biogenesis to function. Development.

[2] Hirakata S., Siomi M.C. (2016). piRNA biogenesis in the germline: From transcription of piRNA genomic sources to piRNA maturation. Biochim. Biophys. Acta.

[3] Czech B., Munafò M., Ciabrelli F., Eastwood E.L., Fabry M.H., Kneuss E., Hannon G.J. (2018). piRNA-Guided Genome Defense: From Biogenesis to Silencing. Annu. Rev. Genet..

[4] Sun Y.H., Lee B., Li X.Z. (2021). The birth of piRNAs: How mammalian piRNAs are produced, originated, and evolved. Mamm. Genome.

[5] Senti K.A., Brennecke J. (2010). The piRNA pathway: A fly’s perspective on the guardian of the genome. Trends Genet..

[6] Muerdter F., Guzzardo P.M., Gillis J., Luo Y., Yu Y., Chen C., Fekete R., Hannon G.J. (2013). A genome-wide RNAi screen draws a genetic framework for transposon control and primary piRNA biogenesis in Drosophila. Mol. Cell.

[7] McClintock B. (1950). The origin and behavior of mutable loci in maize. Proc. Natl. Acad. Sci. USA.

[8] O’Donnell K.A., Boeke J.D. (2007). Mighty Piwis defend the germline against genome intruders. Cell.

[9] Muñoz-Lopez M., Vilar-Astasio R., Tristan-Ramos P., Lopez-Ruiz C., Garcia-Pérez J.L. (2016). Study of Transposable Elements and Their Genomic Impact. Methods Mol. Biol..

[10] Girard A., Sachidanandam R., Hannon G.J., Carmell M.A. (2006). A germline-specific class of small RNAs binds mammalian Piwi proteins. Nature.

[11] Ozata D.M., Gainetdinov I., Zoch A., O’Carroll D., Zamore P.D. (2019). PIWI-interacting RNAs: Small RNAs with big functions. Nat. Rev. Genet..

[12] Iwasaki Y.W., Siomi M.C., Siomi H. (2015). PIWI-interacting RNA: Its biogenesis and functions. Annu. Rev. Biochem..

[13] Goh W.S.S., Falciatori I., Tam O.H., Burgess R., Meikar O., Kotaja N., Hammell M., Hannon G.J. (2015). piRNA-directed cleavage of meiotic transcripts regulates spermatogenesis. Genes Dev..

[14] Zhang P., Kang J.-Y., Gou L.-T., Wang J., Xue Y., Skogerboe G., Dai P., Huang D.-W., Chen R., Fu X.-D. (2015). MIWI and piRNA-mediated cleavage of messenger RNAs in mouse testes. Cell Res..

[15] Dai P., Wang X., Gou L.-T., Li Z.-T., Wen Z., Chen Z.-G., Hua M.-M., Zhong A., Wang L., Su H. (2019). A translation-activating function of MIWI/piRNA during mouse spermiogenesis. Cell.

[16] Zheng K., Wang P.J. (2012). Blockade of pachytene piRNA biogenesis reveals a novel requirement for maintaining post-meiotic germline genome integrity. PLoS Genet..

[17] Özata D.M., Yu T., Mou H., Gainetdinov I., Colpan C., Cecchini K., Kaymaz Y., Wu P.H., Fan K., Kucukural A. (2020). Evolutionarily conserved pachytene piRNA loci are highly divergent among modern humans. Nat. Ecol. Evol..

[18] Gainetdinov I., Colpan C., Arif A., Cecchini K., Zamore P.D. (2018). A single mechanism of biogenesis, initiated and directed by PIWI proteins, explains piRNA production in most animals. Mol. Cell.

[19] Robine N., Lau N.C., Balla S., Jin Z., Okamura K., Kuramochi-Miyagawa S., Blower M.D., Lai E.C. (2009). A broadly conserved pathway generates 3′ UTR-directed primary piRNAs. Curr. Biol..

[20] Aravin A., Gaidatzis D., Pfeffer S., Lagos-Quintana M., Landgraf P., Iovino N., Morris P., Brownstein M.J., Kuramochi-Miyagawa S., Nakano T. (2006). A novel class of small RNAs bind to MILI protein in mouse testes. Nature.

[21] Gou L.-T., Dai P., Yang J.-H., Xue Y., Hu Y.-P., Zhou Y., Kang J.-Y., Wang X., Li H., Hua M.-M. (2014). Pachytene piRNAs instruct massive mRNA elimination during late spermiogenesis. Cell Res..

[22] Saito K., Inagaki S., Mituyama T., Kawamura Y., Ono Y., Sakota E., Kotani H., Asai K., Siomi H., Siomi M.C. (2009). A regulatory circuit for piwi by the large Maf gene traffic jam in Drosophila. Nature.

[23] Klattenhoff C., Xi H., Li C., Lee S., Xu J., Khurana J.S., Zhang F., Schultz N., Koppetsch B.S., Nowosielska A. (2009). The Drosophila HP1 homolog Rhino is required for transposon silencing and piRNA production by dual-strand clusters. Cell.

[24] Malone C.D., Brennecke J., Dus M., Stark A., McCombie W.R., Sachidanandam R., Hannon G.J. (2009). Specialized piRNA pathways act in germline and somatic tissues of the Drosophila ovary. Cell.

[25] Théron E., Dennis C., Brasset E., Vaury C. (2014). Distinct features of the piRNA pathway in somatic and germ cells: From piRNA cluster transcription to piRNA processing and amplification. Mob. DNA.

[26] Ross R.J., Weiner M.M., Lin H. (2014). PIWI proteins and PIWI-interacting RNAs in the soma. Nature.

[27] Rinkevich Y., Rosner A., Rabinowitz C., Lapidot Z., Moiseeva E., Rinkevich B. (2010). Piwi positive cells that line the vasculature epithelium, underlie whole body regeneration in a basal chordate. Dev. Biol..

[28] Martinez V.D., Vucic E.A., Thu K.L., Hubaux R., Enfield K.S., Pikor L.A., Becker-Santos D.D., Brown C.J., Lam S., Lam W.L. (2015). Unique somatic and malignant expression patterns implicate PIWI-interacting RNAs in cancer-type specific biology. Sci. Rep..

[29] Halbach R., Miesen P., Joosten J., Taşköprü E., Rondeel I., Pennings B., Vogels C.B., Merkling S.H., Koenraadt C.J., Lambrechts L. (2020). A satellite repeat-derived piRNA controls embryonic development of Aedes. Nature.

[30] Kim K.W. (2019). PIWI Proteins and piRNAs in the Nervous System. Mol. Cells.

[31] Perrat P.N., DasGupta S., Wang J., Theurkauf W., Weng Z., Rosbash M., Waddell S. (2013). Transposition-driven genomic heterogeneity in the Drosophila brain. Science.

[32] Faulkner G.J., Garcia-Perez J.L. (2017). L1 mosaicism in mammals: Extent, effects, and evolution. Trends Genet..

[33] Larsen P.A., Hunnicutt K.E., Larsen R.J., Yoder A.D., Saunders A.M. (2018). Warning SINEs: Alu elements, evolution of the human brain, and the spectrum of neurological disease. Chromosome Res..

[34] Olivieri D., Sykora M.M., Sachidanandam R., Mechtler K., Brennecke J. (2010). An in vivo RNAi assay identifies major genetic and cellular requirements for primary piRNA biogenesis in Drosophila. EMBO J..

[35] Peng J.C., Lin H. (2013). Beyond transposons: The epigenetic and somatic functions of the Piwi-piRNA mechanism. Curr. Opin. Cell Biol..

[36] Ishizu H., Iwasaki Y.W., Hirakata S., Ozaki H., Iwasaki W., Siomi H., Siomi M.C. (2015). Somatic Primary piRNA Biogenesis Driven by cis-Acting RNA Elements and trans-Acting Yb. Cell Rep..

[37] Kim K.W., Tang N.H., Andrusiak M.G., Wu Z., Chisholm A.D., Jin Y. (2018). A Neuronal piRNA Pathway Inhibits Axon Regeneration in *C. elegans*. Neuron.

[38] Lee D., Yang H., Kim J., Brady S., Zdraljevic S., Zamanian M., Kim H., Paik Y.K., Kruglyak L., Andersen E.C. (2017). The genetic basis of natural variation in a phoretic behavior. Nat. Commun..

[39] Rajasethupathy P., Antonov I., Sheridan R., Frey S., Sander C., Tuschl T., Kandel E.R. (2012). A role for neuronal piRNAs in the epigenetic control of memory-related synaptic plasticity. Cell.

[40] Leighton L.J., Wei W., Marshall P.R., Ratnu V.S., Li X., Zajaczkowski E.L., Spadaro P.A., Khandelwal N., Kumar A., Bredy T.W. (2019). Disrupting the hippocampal Piwi pathway enhances contextual fear memory in mice. Neurobiol. Learn. Mem.

[41] Nandi S., Chandramohan D., Fioriti L., Melnick A.M., Hébert J.M., Mason C.E., Rajasethupathy P., Kandel E.R. (2016). Roles for small noncoding RNAs in silencing of retrotransposons in the mammalian brain. Proc. Natl. Acad. Sci. USA.

[42] Lee E.J., Banerjee S., Zhou H., Jammalamadaka A., Arcila M., Manjunath B., Kosik K.S. (2011). Identification of piRNAs in the central nervous system. RNA.

[43] Kalia L.V., Lang A.E. (2015). Parkinson’s disease. Lancet.

[44] Bloem B.R., Okun M.S., Klein C. (2021). Parkinson’s disease. Lancet.

[45] Bernheimer H., Birkmayer W., Hornykiewicz O., Jellinger K., Seitelberger F. (1973). Brain dopamine and the syndromes of Parkinson and Huntington. Clinical, morphological and neurochemical correlations. J. Neurol. Sci..

[46] Belvisi D., Pellicciari R., Fabbrini A., Costanzo M., Pietracupa S., De Lucia M., Modugno N., Magrinelli F., Dallocchio C., Ercoli T. (2020). Risk factors of Parkinson disease: Simultaneous assessment, interactions, and etiologic subtypes. Neurology.

[47] Moustafa A.A., Chakravarthy S., Phillips J.R., Gupta A., Keri S., Polner B., Frank M.J., Jahanshahi M. (2016). Motor symptoms in Parkinson’s disease: A unified framework. Neurosci. Biobehav. Rev..

[48] Schapira A.H.V., Chaudhuri K.R., Jenner P. (2017). Non-motor features of Parkinson disease. Nat. Rev. Neurosci..

[49] Solla P., Masala C., Pinna I., Ercoli T., Loy F., Orofino G., Fadda L., Defazio G. (2021). Frequency and Determinants of Olfactory Hallucinations in Parkinson’s Disease Patients. Brain Sci..

[50] Dickson D.W. (2012). Parkinson’s disease and parkinsonism: Neuropathology. Cold Spring Harb. Perspect. Med..

[51] Braak H., Ghebremedhin E., Rüb U., Bratzke H., Del Tredici K. (2004). Stages in the development of Parkinson’s disease-related pathology. Cell Tissue Res..

[52] Wakabayashi K., Tanji K., Odagiri S., Miki Y., Mori F., Takahashi H. (2013). The Lewy body in Parkinson’s disease and related neurodegenerative disorders. Mol. Neurobiol..

[53] Gibb W.R., Lees A.J. (1988). The relevance of the Lewy body to the pathogenesis of idiopathic Parkinson’s disease. J. Neurol. Neurosurg. Psychiatry.

[54] Trojanowski J.Q., Lee V.M. (1998). Aggregation of neurofilament and alpha-synuclein proteins in Lewy bodies: Implications for the pathogenesis of Parkinson disease and Lewy body dementia. Arch. Neurol..

[55] Spillantini M.G., Crowther R.A., Jakes R., Hasegawa M., Goedert M. (1998). alpha-Synuclein in filamentous inclusions of Lewy bodies from Parkinson’s disease and dementia with lewy bodies. Proc. Natl. Acad. Sci. USA.

[56] Hou X., Fiesel F.C., Truban D., Castanedes Casey M., Lin W.L., Soto A.I., Tacik P., Rousseau L.G., Diehl N.N., Heckman M.G. (2018). Age- and disease-dependent increase of the mitophagy marker phospho-ubiquitin in normal aging and Lewy body disease. Autophagy.

[57] Minami A., Nakanishi A., Matsuda S., Kitagishi Y., Ogura Y. (2015). Function of α-synuclein and PINK1 in Lewy body dementia (Review). Int. J. Mol. Med..

[58] Beyer K., Domingo-Sàbat M., Ariza A. (2009). Molecular pathology of Lewy body diseases. Int. J. Mol. Sci..

[59] King E., Thomas A. (2017). Systemic Inflammation in Lewy Body Diseases: A Systematic Review. Alzheimer Dis. Assoc. Disord..

[60] Lazdon E., Stolero N., Frenkel D. (2020). Microglia and Parkinson’s disease: Footprints to pathology. J. Neural. Transm..

[61] Schulze M., Sommer A., Plötz S., Farrell M., Winner B., Grosch J., Winkler J., Riemenschneider M.J. (2018). Sporadic Parkinson’s disease derived neuronal cells show disease-specific mRNA and small RNA signatures with abundant deregulation of piRNAs. Acta Neuropathol. Commun..

[62] Wakisaka K.T., Tanaka R., Hirashima T., Muraoka Y., Azuma Y., Yoshida H., Tokuda T., Asada S., Suda K., Ichiyanagi K. (2019). Novel roles of Drosophila FUS and Aub responsible for piRNA biogenesis in neuronal disorders. Brain Res..

[63] Sun W., Samimi H., Gamez M., Zare H., Frost B. (2018). Pathogenic tau-induced piRNA depletion promotes neuronal death through transposable element dysregulation in neurodegenerative tauopathies. Nat. Neurosci..

[64] Huang X., Wong G. (2021). An old weapon with a new function: PIWI-interacting RNAs in neurodegenerative diseases. Transl. Neurodegener..

[65] Tosar J.P., Rovira C., Cayota A. (2018). Non-coding RNA fragments account for the majority of annotated piRNAs expressed in somatic non-gonadal tissues. Commun. Biol..

[66] Freedman J.E., Gerstein M., Mick E., Rozowsky J., Levy D., Kitchen R., Das S., Shah R., Danielson K., Beaulieu L. (2016). Diverse human extracellular RNAs are widely detected in human plasma. Nat. Commun..

[67] Barrett T., Wilhite S.E., Ledoux P., Evangelista C., Kim I.F., Tomashevsky M., Marshall K.A., Phillippy K.H., Sherman P.M., Holko M. (2013). NCBI GEO: Archive for functional genomics data sets—Update. Nucleic Acids Res..

[68] Morin R.D., O’Connor M.D., Griffith M., Kuchenbauer F., Delaney A., Prabhu A.-L., Zhao Y., McDonald H., Zeng T., Hirst M. (2008). Application of massively parallel sequencing to microRNA profiling and discovery in human embryonic stem cells. Genome Res..

[69] Ha H., Song J., Wang S., Kapusta A., Feschotte C., Chen K.C., Xing J. (2014). A comprehensive analysis of piRNAs from adult human testis and their relationship with genes and mobile elements. BMC Genom..

[70] Xu H., Wang X., Wang Z., Li J., Xu Z., Miao M., Chen G., Lei X., Wu J., Shi H. (2020). MicroRNA expression profile analysis in sperm reveals hsa-mir-191 as an auspicious omen of in vitro fertilization. BMC Genom..

[71] Roovers E.F., Rosenkranz D., Mahdipour M., Han C.-T., He N., de Sousa Lopes S.M.C., van der Westerlaken L.A., Zischler H., Butter F., Roelen B.A. (2015). Piwi proteins and piRNAs in mammalian oocytes and early embryos. Cell Rep..

[72] Ryvkin P., Leung Y.Y., Silverman I.M., Childress M., Valladares O., Dragomir I., Gregory B.D., Wang L.-S. (2013). HAMR: High-throughput annotation of modified ribonucleotides. RNA.

[73] Lin J., Wu Y.J., Liang X., Ji M., Ying H.M., Wang X.Y., Sun X., Shao C.H., Zhan L.X., Zhang Y. (2019). Network-based integration of mRNA and miRNA profiles reveals new target genes involved in pancreatic cancer. Mol. Carcinog..

[74] Bratus-Neuenschwander A., Castro-Giner F., Frank-Bertoncelj M., Aluri S., Fucentese S.F., Schlapbach R., Sprott H. (2018). Pain-associated transcriptome changes in synovium of knee osteoarthritis patients. Genes.

[75] Yamane D., Selitsky S.R., Shimakami T., Li Y., Zhou M., Honda M., Sethupathy P., Lemon S.M. (2017). Differential hepatitis C virus RNA target site selection and host factor activities of naturally occurring miR-122 3’ variants. Nucleic Acids Res..

[76] Hoss A.G., Labadorf A., Beach T.G., Latourelle J.C., Myers R.H. (2016). microRNA profiles in Parkinson’s disease prefrontal cortex. Front. Aging Neurosci..

[77] Hoss A.G., Labadorf A., Latourelle J.C., Kartha V.K., Hadzi T.C., Gusella J.F., MacDonald M.E., Chen J.-F., Akbarian S., Weng Z. (2015). miR-10b-5p expression in Huntington’s disease brain relates to age of onset and the extent of striatal involvement. BMC Med. Genom..

[78] Wake C., Labadorf A., Dumitriu A., Hoss A.G., Bregu J., Albrecht K.H., DeStefano A.L., Myers R.H. (2016). Novel microRNA discovery using small RNA sequencing in post-mortem human brain. BMC Genom..

[79] Pantano L., Friedlaender M.R., Escaramis G., Lizano E., Pallares-Albanell J., Ferrer I., Estivill X., Martí E. (2016). Specific small-RNA signatures in the amygdala at premotor and motor stages of Parkinson’s disease revealed by deep sequencing analysis. Bioinformatics.

[80] Pantano L., Pantano F., Marti E., Sui S.H. (2019). Visualization of the small RNA transcriptome using seqclusterViz. F1000Research.

[81] Sproviero D., Gagliardi S., Zucca S., Arigoni M., Giannini M., Garofalo M., Olivero M., Dell’Orco M., Pansarasa O., Bernuzzi S. (2021). Different miRNA Profiles in Plasma Derived Small and Large Extracellular Vesicles from Patients with Neurodegenerative Diseases. Int. J. Mol. Sci..

[82] Martin M. (2011). Cutadapt removes adapter sequences from high-throughput sequencing reads. EMBnet. J..

[83] Yates A.D., Achuthan P., Akanni W., Allen J., Allen J., Alvarez-Jarreta J., Amode M.R., Armean I.M., Azov A.G., Bennett R. (2020). Ensembl 2020. Nucleic Acids Res..

[84] Langmead B. (2010). Aligning short sequencing reads with Bowtie. Curr. Protoc. Bioinform..

[85] Lee C.M., Barber G.P., Casper J., Clawson H., Diekhans M., Gonzalez J.N., Hinrichs A.S., Lee B.T., Nassar L.R., Powell C.C. (2020). UCSC Genome Browser enters 20th year. Nucleic Acids Res..

[86] O’Leary N.A., Wright M.W., Brister J.R., Ciufo S., Haddad D., McVeigh R., Rajput B., Robbertse B., Smith-White B., Ako-Adjei D. (2016). Reference sequence (RefSeq) database at NCBI: Current status, taxonomic expansion, and functional annotation. Nucleic Acids Res..

[87] Frankish A., Diekhans M., Ferreira A.-M., Johnson R., Jungreis I., Loveland J., Mudge J.M., Sisu C., Wright J., Armstrong J. (2019). GENCODE reference annotation for the human and mouse genomes. Nucleic Acids Res..

[88] Leung Y.Y., Kuksa P.P., Amlie-Wolf A., Valladares O., Ungar L.H., Kannan S., Gregory B.D., Wang L.-S. (2016). DASHR: Database of small human noncoding RNAs. Nucleic Acids Res..

[89] Liao Y., Smyth G.K., Shi W. (2014). featureCounts: An efficient general purpose program for assigning sequence reads to genomic features. Bioinformatics.

[90] Wang J., Zhang P., Lu Y., Li Y., Zheng Y., Kan Y., Chen R., He S. (2019). piRBase: A comprehensive database of piRNA sequences. Nucleic Acids Res..

[91] Fernandes J.D., Zamudio-Hurtado A., Clawson H., Kent W.J., Haussler D., Salama S.R., Haeussler M. (2020). The UCSC repeat browser allows discovery and visualization of evolutionary conflict across repeat families. Mob. DNA.

[92] Love M.I., Huber W., Anders S. (2014). Moderated estimation of fold change and dispersion for RNA-seq data with DESeq2. Genome Biol..

[93] Stein C.K., Qu P., Epstein J., Buros A., Rosenthal A., Crowley J., Morgan G., Barlogie B. (2015). Removing batch effects from purified plasma cell gene expression microarrays with modified ComBat. BMC Bioinform..

[94] Raudvere U., Kolberg L., Kuzmin I., Arak T., Adler P., Peterson H., Vilo J. (2019). g:Profiler: A web server for functional enrichment analysis and conversions of gene lists (2019 update). Nucleic Acids Res..

[95] Ståhle L., Wold S. (1987). Partial least squares analysis with cross-validation for the two-class problem: A Monte Carlo study. J. Chemom..

[96] Barker M., Rayens W. (2003). Partial least squares for discrimination. J. Chemom. J. Chemom. Soc..

[97] Lê Cao K.-A., Boitard S., Besse P. (2011). Sparse PLS discriminant analysis: Biologically relevant feature selection and graphical displays for multiclass problems. BMC Bioinform..

[98] Stone M. (1974). Cross-validatory choice and assessment of statistical predictions. J. R. Stat. Soc. Ser. B.

[99] Allen D.M. (1974). The relationship between variable selection and data agumentation and a method for prediction. Technometrics.

[100] Hanley J.A., McNeil B.J. (1982). The meaning and use of the area under a receiver operating characteristic (ROC) curve. Radiology.

[101] Rohart F., Gautier B., Singh A., Lê Cao K.-A. (2017). mixOmics: An R package for ‘omics feature selection and multiple data integration. PLoS Comput. Biol..

[102] Yan Z., Hu H.Y., Jiang X., Maierhofer V., Neb E., He L., Hu Y., Hu H., Li N., Chen W. (2011). Widespread expression of piRNA-like molecules in somatic tissues. Nucleic Acids Res..

[103] Carmell M.A., Girard A., van de Kant H.J., Bourc’his D., Bestor T.H., de Rooij D.G., Hannon G.J. (2007). MIWI2 is essential for spermatogenesis and repression of transposons in the mouse male germline. Dev. Cell.

[104] Perera B.P., Tsai Z.T.-Y., Colwell M.L., Jones T.R., Goodrich J.M., Wang K., Sartor M.A., Faulk C., Dolinoy D.C. (2019). Somatic expression of piRNA and associated machinery in the mouse identifies short, tissue-specific piRNA. Epigenetics.

[105] Faulkner G.J., Kimura Y., Daub C.O., Wani S., Plessy C., Irvine K.M., Schroder K., Cloonan N., Steptoe A.L., Lassmann T. (2009). The regulated retrotransposon transcriptome of mammalian cells. Nat. Genet..

[106] Watanabe T., Cheng E.-c., Zhong M., Lin H. (2015). Retrotransposons and pseudogenes regulate mRNAs and lncRNAs via the piRNA pathway in the germline. Genome Res..

[107] Wang C., Lin H. (2021). Roles of piRNAs in transposon and pseudogene regulation of germline mRNAs and lncRNAs. Genome Biol..

[108] Uhrig S., Klein H. (2019). PingPongPro: A tool for the detection of piRNA-mediated transposon-silencing in small RNA-Seq data. Bioinformatics.

[109] Zhang D., Tu S., Stubna M., Wu W.-S., Huang W.-C., Weng Z., Lee H.-C. (2018). The piRNA targeting rules and the resistance to piRNA silencing in endogenous genes. Science.

[110] Chalorak P., Dharmasaroja P., Meemon K. (2020). Downregulation of eEF1A/EFT3-4 Enhances Dopaminergic Neurodegeneration After 6-OHDA Exposure in C. elegans Model. Front. Neurosci..

[111] Dumitriu A., Golji J., Labadorf A.T., Gao B., Beach T.G., Myers R.H., Longo K.A., Latourelle J.C. (2015). Integrative analyses of proteomics and RNA transcriptomics implicate mitochondrial processes, protein folding pathways and GWAS loci in Parkinson disease. BMC Med Genom..

[112] Shen E.-Z., Chen H., Ozturk A.R., Tu S., Shirayama M., Tang W., Ding Y.-H., Dai S.-Y., Weng Z., Mello C.C. (2018). Identification of piRNA binding sites reveals the argonaute regulatory landscape of the C. elegans germline. Cell.

[113] Chavda V., Madhwani K., Chaurasia B. (2021). PiWi RNA in Neurodevelopment and Neurodegenerative disorders. Curr. Mol. Pharmacol..

[114] Wakisaka K.T., Imai Y. (2019). The dawn of pirna research in various neuronal disorders. Front. Biosci. (Landmark Ed.).

[115] Hyun S. (2017). Small RNA Pathways That Protect the Somatic Genome. Int. J. Mol. Sci..

[116] Reik W. (2007). Stability and flexibility of epigenetic gene regulation in mammalian development. Nature.

[117] Barckmann B., Barckmann B., Pierson S., Dufourt J., Papin C., Armenise C., Port F., Grentzinger T., Chambeyron S., Baronian G. (2015). Aubergine iCLIP reveals piRNA-dependent decay of mRNAs involved in germ cell development in the early embryo. Cell Rep..

[118] Rojas-Ríos P., Chartier A., Pierson S., Simonelig M. (2017). Aubergine and piRNAs promote germline stem cell self-renewal by repressing the proto-oncogene Cbl. EMBO J..

[119] Tang W., Seth M., Tu S., Shen E.-Z., Li Q., Shirayama M., Weng Z., Mello C.C. (2018). A sex chromosome piRNA promotes robust dosage compensation and sex determination in C. elegans. Dev. Cell.

[120] Balaratnam S., West N., Basu S. (2018). A piRNA utilizes HILI and HIWI2 mediated pathway to down-regulate ferritin heavy chain 1 mRNA in human somatic cells. Nucleic Acids Res..

[121] Wu P.-H., Fu Y., Cecchini K., Özata D.M., Arif A., Yu T., Colpan C., Gainetdinov I., Weng Z., Zamore P.D. (2020). The evolutionarily conserved piRNA-producing locus pi6 is required for male mouse fertility. Nat. Genet..

[122] Li X.Z., Roy C.K., Dong X., Bolcun-Filas E., Wang J., Han B.W., Xu J., Moore M.J., Schimenti J.C., Weng Z. (2013). An ancient transcription factor initiates the burst of piRNA production during early meiosis in mouse testes. Mol. Cell.

[123] Fu A., Jacobs D.I., Zhu Y. (2014). Epigenome-wide analysis of piRNAs in gene-specific DNA methylation. RNA Biol..

[124] Dufourt J., Bontonou G., Chartier A., Jahan C., Meunier A.-C., Pierson S., Harrison P.F., Papin C., Beilharz T.H., Simonelig M. (2017). piRNAs and Aubergine cooperate with Wispy poly (A) polymerase to stabilize mRNAs in the germ plasm. Nat. Commun..

[125] Vourekas A., Alexiou P., Vrettos N., Maragkakis M., Mourelatos Z. (2016). Sequence-dependent but not sequence-specific piRNA adhesion traps mRNAs to the germ plasm. Nature.

[126] Klein J.D., Qu C., Yang X., Fan Y., Tang C., Peng J.C. (2016). c-Fos repression by Piwi regulates Drosophila ovarian germline formation and tissue morphogenesis. PLoS Genet..

[127] Braak H., Braak E. (2000). Pathoanatomy of Parkinson’s disease. J. Neurol..

[128] Cools R., Clark L., Owen A.M., Robbins T.W. (2002). Defining the neural mechanisms of probabilistic reversal learning using event-related functional magnetic resonance imaging. J. Neurosci..

[129] Vriend C., Boedhoe P.S., Rutten S., Berendse H.W., van der Werf Y.D., van den Heuvel O.A. (2016). A smaller amygdala is associated with anxiety in Parkinson’s disease: A combined FreeSurfer—VBM study. J. Neurol. Neurosurg. Psychiatry.

[130] Carey G., Görmezoğlu M., de Jong J.J.A., Hofman P.A.M., Backes W.H., Dujardin K., Leentjens A.F.G. (2021). Neuroimaging of Anxiety in Parkinson’s Disease: A Systematic Review. Mov. Disord..

[131] Izadpanah M., Seddigh A., Ebrahimi Barough S., Fazeli S.A.S., Ai J. (2018). Potential of Extracellular Vesicles in Neurodegenerative Diseases: Diagnostic and Therapeutic Indications. J. Mol. Neurosci..

[132] Gámez-Valero A., Beyer K., Borràs F.E. (2019). Extracellular vesicles, new actors in the search for biomarkers of dementias. Neurobiol. Aging.

[133] Lamontagne-Proulx J., St-Amour I., Labib R., Pilon J., Denis H.L., Cloutier N., Roux-Dalvai F., Vincent A.T., Mason S.L., Williams-Gray C. (2019). Portrait of blood-derived extracellular vesicles in patients with Parkinson’s disease. Neurobiol. Dis..

[134] Ohmichi T., Mitsuhashi M., Tatebe H., Kasai T., Ali El-Agnaf O.M., Tokuda T. (2019). Quantification of brain-derived extracellular vesicles in plasma as a biomarker to diagnose Parkinson’s and related diseases. Parkinsonism Relat. Disord..

[135] Leggio L., Paternò G., Vivarelli S., Falzone G.G., Giachino C., Marchetti B., Iraci N. (2021). Extracellular Vesicles as Novel Diagnostic and Prognostic Biomarkers for Parkinson’s Disease. Aging Dis..

[136] He S., Huang L., Shao C., Nie T., Xia L., Cui B., Lu F., Zhu L., Chen B., Yang Q. (2021). Several miRNAs derived from serum extracellular vesicles are potential biomarkers for early diagnosis and progression of Parkinson’s disease. Transl. Neurodegener..

[137] Lööv C., Scherzer C.R., Hyman B.T., Breakefield X.O., Ingelsson M. (2016). α-Synuclein in Extracellular Vesicles: Functional Implications and Diagnostic Opportunities. Cell Mol. Neurobiol..

[138] Kitamura Y., Kojima M., Kurosawa T., Sasaki R., Ichihara S., Hiraku Y., Tomimoto H., Murata M., Oikawa S. (2018). Proteomic Profiling of Exosomal Proteins for Blood-based Biomarkers in Parkinson’s Disease. Neuroscience.

[139] Li M.J., Wang P., Liu X., Lim E.L., Wang Z., Yeager M., Wong M.P., Sham P.C., Chanock S.J., Wang J. (2012). GWASdb: A database for human genetic variants identified by genome-wide association studies. Nucleic Acids Res..

[140] Peng Q., Long C.L., Malhotra S., Humphrey M.B. (2013). A physical interaction between the adaptor proteins DOK3 and DAP12 is required to inhibit lipopolysaccharide signaling in macrophages. Sci. Signal.

[141] Qin L., Wu X., Block M.L., Liu Y. (2007). Systemic LPS causes chronic neuroinflammation and progressive neurodegeneration. Glia.

[142] Kim S.R., Kareva T., Yarygina O., Kholodilov N., Burke R.E. (2012). AAV transduction of dopamine neurons with constitutively active Rheb protects from neurodegeneration and mediates axon regrowth. Mol. Ther..

[143] Vanni S., Zattoni M., Moda F., Giaccone G., Tagliavini F., Haïk S., Deslys J.-P., Zanusso G., Ironside J.W., Carmona M. (2018). Hemoglobin mRNA changes in the frontal cortex of patients with neurodegenerative diseases. Front. Neurosci..

[144] Shen L., Wang C., Chen L., Wong G. (2021). Dysregulation of MicroRNAs and PIWI-Interacting RNAs in a Caenorhabditis elegans Parkinson’s Disease Model Overexpressing Human α-Synuclein and Influence of tdp-1. Front. Neurosci..

[145] Stephenson J., Nutma E., van der Valk P., Amor S. (2018). Inflammation in CNS neurodegenerative diseases. Immunology.

[146] Guo C., Jeong H.H., Hsieh Y.C., Klein H.U., Bennett D.A., De Jager P.L., Liu Z., Shulman J.M. (2018). Tau Activates Transposable Elements in Alzheimer’s Disease. Cell Rep..

[147] Ravel-Godreuil C., Znaidi R., Bonnifet T., Joshi R.L., Fuchs J. (2021). Transposable elements as new players in neurodegenerative diseases. FEBS Lett..

