# Supplementary material for: Dysregulation of Human Somatic piRNA Expression in Parkinson’s Disease Subtypes and Stages
Source: Int J Mol Sci. 2022 Feb 23;23(5):2469. doi: 10.3390/ijms23052469 (PMC8910154; doi:10.3390/ijms23052469)
Supplement: Supplementary file 1 [file ijms-23-02469-s001.zip › ijms-1577990-supplementary.pdf]

Supplementary Figure S1

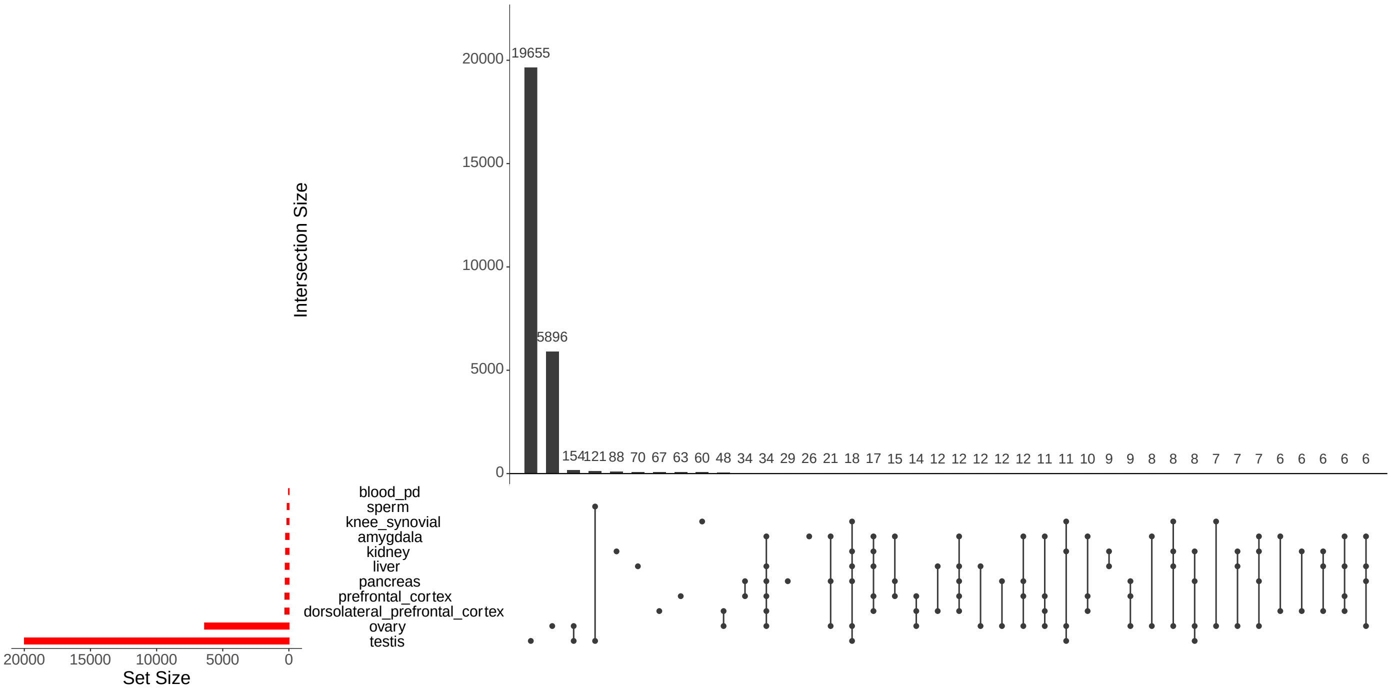

Supplementary Figure S1. Upset plot.

The number of piRNA types among somatic tissues (dorsolateral prefrontal cortex, prefrontal cortex, amygdala, pancreas, liver, knee synovial), germline tissues (testis, sperm, ovary), and brain tissues (dorsolateral prefrontal cortex, prefrontal cortex, amygdala) were plotted.

Supplementary Figure S2

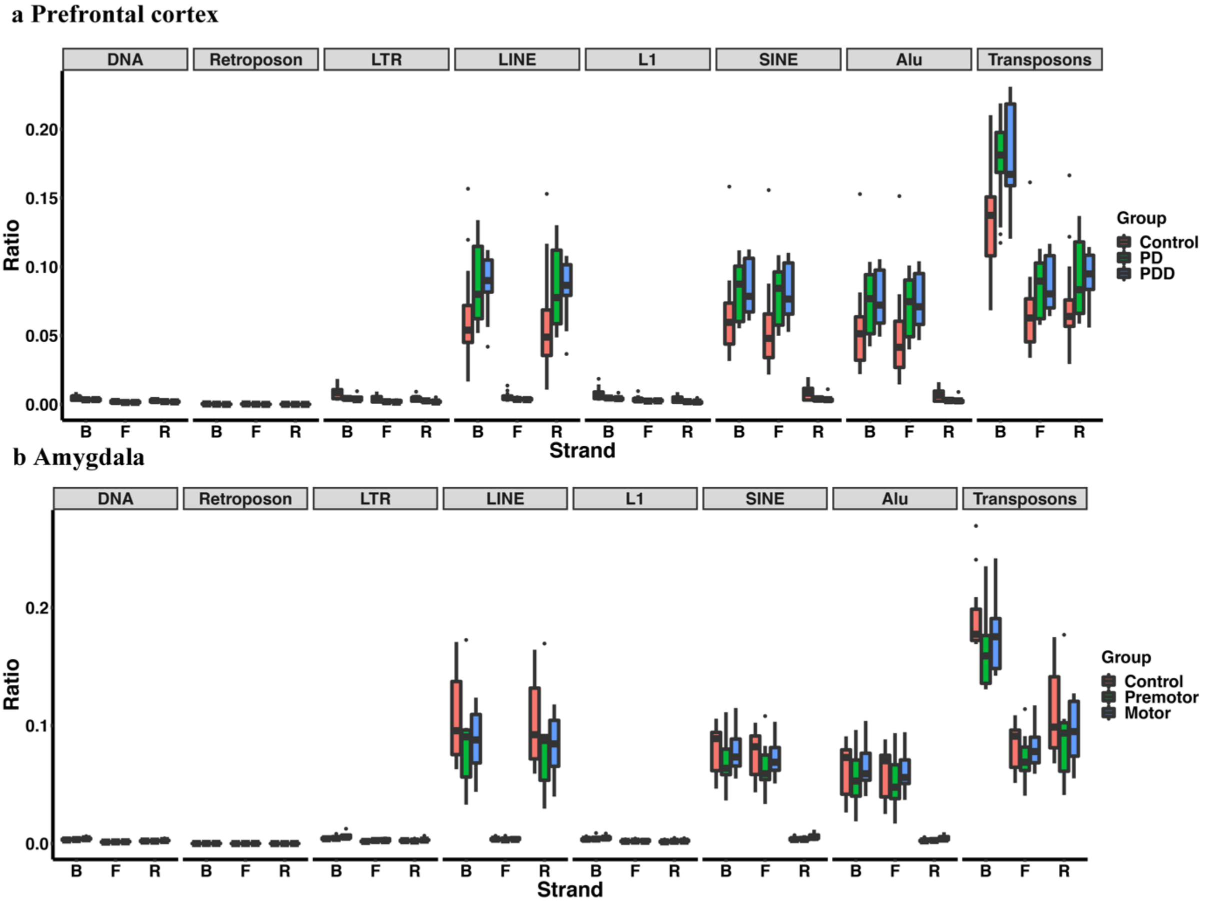

Supplementary Figure S2. Genomic context (transposon) of piRNAs.

The percentage of annotated piRNAs reads mapped to transposons and transposon subtypes in prefrontal cortex **(a)** and amygdala **(b)**. (Ratio = reads number mapped to transposons (or lncRNA, protein-coding genes, pseudogenes) / total annotated piRNAs reads number) are shown. The strand symbols are: B, both; F, forward; R, Reverse.

Supplementary Table S1

sPLS-DA of piRNAs expression data							
	Prefrontal cortex		Amygdala		Blood MV	Blood EXO	Blood MV+EXO
Classification	Control vs PD (PD + PDD)	Control vs PD vs PDD	Control vs PD (premotor + motor)	Control vs premotor vs motor	Control vs PD		Control vs PD
Categories	2	3	2	3	2		2
Number components	7	8	4	6	1		3
Loading	6,9,5,20,5,30,5,7,5	10,5,10,7,60,20,5,9	10.30,5,5	40,50,30,5,20.80	10		5,40,20
R2							
First comp.	0.17	0.16	0.19	0.27	0.24		0.16
Second comp.	0.056	0.062	0.13	0.077			0.17
Third comp.	0.094	0.056	0.13	0.066			0.12
Fourth comp.	0.035	0.051	0.031	0.023			
Fifth comp.	0.095	0.087		0.028			
Sixth comp.	0.056	0.051		0.049			
Seventh comp.	0.019	0.042					
Eighth comp.		0.018					
Nine comp.						Expression level too low	
Train performance	AUC = 1, p-value = 1.37e-09	Control vs Others: AUC = 1 ;P value = 1.37e-09	AUC = 1, p-value = 6.7e-06	Control vs Others: AUC = 1 ;P value = 6.70e-06	AUC = 1; P value = 0.00146		AUC = 1; P value = 0.00146
		PD vs Others: AUC = 1; P value = 9.22e-09		Premotor vs Others: AUC =1; P value = 9.64e-05			
		PDD vs Others: AUC = 1, P value = 3.18e-06		Motor vs Others: AUC = 1, P value = 9.64e-05			
Leave-one-out verification performance	69%; AUC = 0.73	64%	79%; AUC = 0.82	71%	92%; AUC =0.89		72%; AUC = 0.74
		Control: AUC = 0.79		Control: AUC = 0.82			
		PD : AUC = 0.78		Premotor : AUC = 0.84			
		PDD: AUC = 0.69		Motor: AUC = 0.74			

sPLS-DA of piRNAs expression data in gene regions							
	Prefrontal cortex		Amygdala		Blood SEVs	Blood LEVs	Blood SEVs+LEVs
Classification	Control vs PD (PD + PDD)	Control vs PD vs PDD	Control vs PD (premotor + motor)	Control vs premotor vs motor	Control vs PD	Control vs PD	Control vs PD
Categories	2	3	2	3	2	2	2
Number components	2	2	3	6	1	4	1
Loading	5,5	40,5	8,5,5	5,10,30,7,8,20	5	5,2,5,6	90
R2							
First comp.	0.32	0.51	0.29	0.28	0.36	0.14	0.16
Second comp.	0.029	0.035	0.055	0.082		0.18	
Third comp.			0.072	0.089		0.23	
Fourth comp.				0.058		0.13	
Fifth comp.				0.03			
Sixth comp.				0.016			
Seventh comp.							
Eighth comp.							
Nine comp.							

Train performance	AUC = 0.96, p-value = 2.00e-08	Control vs Others: AUC = 0.96 ;P value = 1.79e-08	AUC = 1, pvalue = 6.70e-06	Control vs Others: AUC = 1 ;P value = 6.70e-06	AUC = 1, Pvalue = 0.0015	AUC = 1, P value = 0.0015	AUC = 0.98; P value = 0.0022
		PD vs Others: AUC = 0.89; P value = 7.65e-06		Premotor vs Others: AUC =1; P value = 9.64e-05			
		PDD vs Others: AUC = 0.68, P value = 0.093		Motor vs Others: AUC = 1, P value = 9.64e-05			
Leave-one-out verification performance	77%; AUC = 0.80	50%	71%; AUC = 0.82	64%	83%; AUC = 0.83	69%; AUC = 0.72	83%; AUC = 0.85
		Control: AUC = 0.83		Control: AUC = 0.76			
		PD : AUC = 0.80		Premotor : AUC = 0.81			
		PDD: AUC = 0.45		Motor: AUC = 0.57			

**sPLS-DA of piRNAs expression data in repeats regions**							
	Prefrontal cortex		Amygdala		Blood SEVs	Blood LEVs	Blood SEVs+LEVs
Classification	Control vs PD (PD + PDD)	Control vs PD vs PDD	Control vs PD (premotor + motor)	Control vs premotor vs motor	Control vs PD	Control vs PD	Control vs PD
Categories	2	3	2	3	2	2	2
Number components	1	2	9	4	9	1	3
Loading	100	30,30	6,9,5,20,5,30,5,7,5	30,40,20,6	9,5,5,5,5,6,5,5,5	3	5,5,10
R2							
First comp.	0.58	0.53	0.19	0.26	0.36	0.42	0.18
Second comp.		0.077	0.1	0.058	0.22		0.19
Third comp.			0.12	0.14	0.083		0.11
Fourth comp.			0.059	0.054	0.067		
Fifth comp.			0.079		0.073		
Sixth comp.			0.041		0.039		
Seventh comp.			0.031		0.026		
Eighth comp.			0.034		0.051		
Nine comp.			0.032		0.062		
Train performance	AUC = 0.80, p-value = 3.28e-04	Control vs Others: AUC = 0.91 ;P value = 7.36e-07	AUC = 1, pvalue = 6.70e-06	Control vs Others: AUC = 1 ;P value = 6.70e-06	AUC = 1; P value = 0.0015	AUC = 0.63; P value = 0.41	AUC = 0.98; P value = 0.0022
		PD vs Others: AUC = 0.75; P value = 0.0035		Premotor vs Others: AUC =1; P value = 9.64e-05			
		PDD vs Others:		Motor vs Others:			

		AUC = 0.88, P value = 4.7e-04		AUC = 1, P value = 9.64e-05			
Leave-one-out verification performance	77%; AUC = 0.68	42%	82%; AUC = 0.89	55%	86%; AUC = 0.91	36%; AUC = 0.26	64%; AUC = 0.5
		Control: AUC = 0.69		Control: AUC = 0.83			
		PD : AUC = 0.55		Premotor : AUC = 0.78			
		PDD: AUC = 0.44		Motor: AUC = 0.63			

sPLS-DA of piRNAs expression data in genes+repeats regions							
	Prefrontal cortex		Amygdala		Blood SEVs	Blood LEVs	Blood SEVs+LEVs
Classification	Control vs PD (PD + PDD)	Control vs PD vs PDD	Control vs PD (premotor + motor)	Control vs premotor vs motor	Control vs PD	Control vs PD	Control vs PD
Categories	2	3	2	3	2	2	2
Number components	2	4	3	5	1	10	1
Loading	80,5	60,5,30,80	30,50,100	20,60,40,6,60	60	8,5,7,5,5,9,9,5,5,5	60
R2							
First comp.	0.52	0.5	0.3	0.27	0.28	0.18	0.14
Second comp.	0.0088	0.0085	0.12	0.11		0.21	
Third comp.		0.046	0.034	0.091		0.12	
Fourth comp.		0.097		0.023		0.091	
Fifth comp.				0.067		0.14	
Sixth comp.						0.082	
Seventh comp.						0.067	
Eighth comp.						0.036	
Nine comp.						0.031	
Tenth comp.						0.022	
Train performance	AUC = 0.97, p-value = 1.60e-08	Control vs Others: AUC = 0.99 ;P value = 2.82e-09	AUC = 1, pvalue = 6.70e-06	Control vs Others: AUC = 1 ;P value = 6.70e-06	AUC = 1; P value = 0.0015	AUC = 1; P value = 0.0015	AUC = 1; P value = 0.0015
		PD vs Others: AUC = 0.99; P value = 1.89e-08		Premotor vs Others: AUC =1; P value = 9.64e-05			
		PDD vs Others: AUC = 0.99, P value = 4.58e-06		Motor vs Others: AUC = 1, P value = 9.64e-05			
Leave-one-out verification performance	84%; AUC = 0.87	59%	71%; AUC = 0.82	71%	78%; AUC = 0.70	75%; AUC = 0.81	69%; AUC = 0.76
		Control: AUC = 0.86		Control: AUC = 0.84			

		PD : AUC = 0.71		Premotor : AUC = 0.56			
		PDD: AUC = 0.76		Motor: AUC = 0.88			

Supplementary Table S1. PLS-DA of piRNAs expression data.

The R2 value provides the variability explained by each component. Red text marks the classifier with the best performance within each tissue (prefrontal cortex, amygdala, blood LEVs, blood SEVs, blood LEVs+ SEVs) or test (PD vs control, PD vs PDD vs control, PD vs premotor vs motor stage).
